# The impact of the great economic crisis on mental health care in Italy

**DOI:** 10.1007/s10198-020-01204-w

**Published:** 2020-06-13

**Authors:** Yuxi Wang, Giovanni Fattore

**Affiliations:** Centre for Research on Health and Social Care Management Department of Social and Political Science, Bocconi University, 3-C1-01 Via Guglielmo Röntgen, 1, 20136 Milano, MI Italy

**Keywords:** Mental illness, Mental health care, Economic crisis, Unemployment, I14, I15, E24

## Abstract

The great economic crisis in 2008 has affected the welfare of the population in countries such as Italy. Although there is abundant literature on the impact of the crisis on physical health, very few studies have focused on the causal implications for mental health and health care. This paper, therefore, investigates the impact of the recent economic crisis on hospital admissions for severe mental disorder at small geographic levels in Italy and assesses whether there are heterogeneous effects across areas with distinct levels of income. We exploit 9-year (2007–2015) panel data on hospital discharges, which is merged with employment and income composition at the geographic units that share similar labour market structures. Linear and dynamic panel analysis are used to identify the causal effect of rising unemployment rate on severe mental illness admissions per 100,000 residents to account for time-invariant heterogeneity. We further create discrete income levels to identify the potential socioeconomic gradients behind this effect across areas with different economic characteristics. The results show a significant impact of higher unemployment rates on admissions for severe mental disorders after controlling for relevant economic factors, and the effects are concentrated on the most economically disadvantaged areas. The results contribute to the literature of spatio-temporal variation in the broader determinants of mental health and health care utilisation and shed light on the populations that are most susceptible to the effects of the economic crisis.

## Introduction

Studies on the social determinants of mental health date back to the early twentieth century when Faris and Dunham [1] examined the relationship between Chicago area neighbourhood structural characteristics and mental disorder rates. They found high rates of severe mental disorders in disadvantaged neighbourhoods. These results have spearheaded the sociological research interests in the relationship between socioeconomic factors and mental disorder. In the ensuing years, increasing numbers of studies have investigated the variation of mental disorder incidents across areas with different levels of socioeconomic deprivation [2–6]. These cross-sectional studies have all pointed to the intuitive correlation between a higher mental disorder prevalence or psychiatric admission rate and a higher degree of economic deprivation in the neighbourhood.

The advent of the global financial crisis in 2008 and its economic consequences prompted a revival of this stream of literature, which subsequently assesses the relationship between macroeconomic conditions and mental health outcomes. Conceptually, at the individual level, economic crisis can affect mental health through increased unemployment, perceived insecurity, indebtedness, or decreasing welfare support. Many recent studies have documented the prolonged mental health effects of worsening economic conditions. For instance, in Spain, researchers have shown significant associations between crisis periods and increased frequency of primary care mental disorder diagnosis [7] or self-assessed mental health [8]. Similarly, results are found in relation to different types of affective disorder admission or diagnosis [9–12] and self-reported mental health in various European and US studies [13–17]. However, some Spanish studies have contradictory results, as they found the economic crisis to be associated with a lower number of people demanding mental health services [18, 19].

In Italy, the crisis has had profound implications on the population. The systematic rise in unemployment rates and the worsening labour conditions have given rise to substantial inequalities and social tensions [20]. Also, the generally pessimistic outlook of the economy could have posed additional severe mental health challenges due to the widespread insecurity. Moreover, the governments did not use counter-cycle measures but instead implemented austerity measures, and the health care sector was thereby faced with budget cuts to avoid debt default [21]. These fiscal policies may have unintentionally exacerbated unequal access to care across socioeconomic groups and geographic areas, and the consequences on mental health care utilisation of the population remain under-explored. If highly disadvantaged population not only have a greater need for mental health care due to the crisis, but these needs are not met due to inadequate resources being allocated to the corresponding services, equity concerns arise. This unmet need may even aggravate the burden on the health care system in the long run.

Although the literature on the association between economic crisis and mental health and health care utilisation is plentiful, the research in the Italian context has investigated the issue either using longitudinal survey data with subjective measurement of mental health [22] or looking at the correlation between mental disorder and crisis period at the aggregated level [23]. To our knowledge, no study has, at the Italian national level, proved the causal effect of the economic crisis on mental health care. We aim to contribute to this stream of literature by analysing the potential impact of changing economic conditions on mental disorder admissions throughout the crisis period in Italy. We pay special attention to the differential effect of the crisis on areas characterised by high- and low-income levels. As discussed in the following section, there is a marked paucity of studies that established the causal impact of the crisis on mental health care using administrative data. The results will be informative for policymakers in higher–middle- to high-income countries that had experienced rapid socioeconomic changes accompanied by an increasingly cost-conscious health care system.

### Related literature

To establish the socioeconomic determinants of mental health outcomes, we need to look into multi-disciplinary works for deeper understandings of how adverse conditions act as psychological stressors and how such conditions can have implications on the health care system. While the biological or psychological process is beyond the scope of this paper, we intend to invoke social science theories at the micro-social and macro-social levels to explain this link.

The psychological effects of living around neighbourhoods characterised by low social status are explored in the early literature [24, 25]. The emphasis is primarily on the social causes of psychological stress, including the amount of control and autonomy over the environment a person resides [26], the extent to which one feels adequately rewarded for the labour [27–29], or deprivation in its various forms. We recognise the importance of the psycho-social factors, but given our empirical interest, we will only discuss the economic explanation in greater length. Blane [30] identified the materialist explanation for psychological stress as the “experience arising as a consequence of social structure and organisation, over which the individual has no control”. This illustration is linked to Weber [31] ’s concept of “life chances”, which depends on one’s bargaining power in the labour market [32]. The feeling of little control and of being trapped can evoke frustration and anxiety[33]. This response is likely to happen if individuals from a deprived condition have no means or qualifications to obtain jobs, and the disadvantage is likely to be exacerbated by the neighbourhood where one resides.

At the community level, theories on the sociological process that creates neighbourhood disorders focus on stressors and their implications on residents’ health and wellbeing [34–36]. As discussed above, the lack of control and autonomy can contribute to the variation of health across social gradients [37]. Residents who experience concentrated deprivation can generate a widespread sense of powerlessness and mistrust, which can further lead to psychological distress—anxiety, anger and depression [38]. At the macro-societal level, theories on the loss of control during socioeconomic transitions provide insights into the mechanism behind the impact on health. Instability and insecurity in the labour market and unemployment during economic transitions or economic shocks can contribute to the rise in psychological and somatic responses such as chronic stress and anxiety [39]. Lower levels of perceived agency can diminish optimism for the future and ultimately result in poorer population health [38]. These broader adverse conditions can activate the chronic arousal of the stress system in its pathway to influence one’s mental health [40]. In the established theoretical literature, area-level socioeconomic factors are indisputably fundamental causes of mental illness.

Social epidemiologists and psychiatric scientists have long investigated the socioeconomic and environmental determinants of mental illnesses empirically. The early study by Faris and Dunham [1] examined the geographic distribution of mental disorders across economic gradients. Their systematic analysis pioneered future studies on the association between social disorganisation and mental disorder [2, 41–44]. These studies tested correlations between psychiatric admissions and socioeconomic indicators of the neighbourhood, showing a non-homogenous distribution of admissions to psychiatric care and mental disorders across areas that are differentially deprived.

Interests in this field of research resurfaced with the advent of the great economic crisis, during which rising unemployment and deteriorating working conditions have had implications on the population’s mental health. While most research in the economics literature have analysed physical health outcomes and utilisation [17, 45–48], there is much less understanding on the impact of macroeconomic conditions on mental health and health care. Ruhm [49] summarised the previous research and broadly concluded that total mortality is pro-cyclical, that death increases during an economic boom, while for the sub-category of suicides or intentional self-harm the relation can be counter-cyclical. Among other related studies, Belloni et al. [50] have shown that mental health improves upon retirement among 10 European countries, especially for regions that are hit severely by the economic crisis; Drydakis [16] found more devastating effects of unemployment on mental health during the crisis in Greece; McInerney and Mellor [51] have found that sudden wealth loss due to the 2008 market crash caused immediate decline in mental health. Most of the research utilised subjective measures of mental health.

Systematic reviews from inter-disciplinary research provided ample evidence on how economic recessions can be associated with mental health outcome and utilisation [52–54]. Frasquilho et al. [52] found that economic indicators such as rising unemployment and declining income are significantly associated with poor mental wellbeing and increased rates of mental disorders. The majority of the studies investigated countries that are hit the hardest by the economic recession such as Greece [9, 10], Spain [7, 8, 55–57] and Italy [22, 58–60], though primarily using cross-sectional surveys or ecological analysis, thus providing limited evidence of causal inferences [52]. Parmar et al. [53] identified relatively consistent results on the association between deteriorating economic conditions and poor mental health, although risks of bias persist in the studies due to selection and potential confounding effects. A recent systematic review by Silva et al. [54] further summarised the empirical evidence on the association between periods of economic crisis and the use of mental health care, suggesting that periods of economic crisis can be linked to an increase in hospital admissions for mental disorders. For instance, a cohort study by Modrek et al. [11] found a marginally significant increase in the post-recession trend in inpatient utilisation compared with pre-recession trend in the US, while Lee et al. [61], in a time series analysis, found increased hospitalisation rate for affective disorders in Taiwan, especially among the low-income group. We aim to further investigate the causal impact of changing economic conditions on mental health and health care and the social gradient behind in the Italian context, given that the indirect costs in the form of lost mental capital and productivity can pose major challenges for the society.

Another factor related to the economic determinants of mental health is the role of income inequality. The earliest papers on physical health and income inequality showed a cross-sectional association between Gini coefficients of income inequality and various health outcomes [62, 63]. The literature rapidly expanded in early 2000, and a review by Wilkinson and Pickett [64] showed an overwhelming majority of the studies found a positive relationship between income inequality and health. As the gulf between the poor and the rich widens in recent decades, many scholars explicitly looked into the effect of inequality on mental health. A 2017 Lancet Psychiatry meta-analysis collected data from 27 eligible studies and showed that there is a systematic negative effect of income inequality on mental health, with effects that vary widely across countries [65]. Most recently, an in-depth examination illustrated how vast disparities of wealth are associated with elevated levels of stress, anxiety and ultimately, depression and bipolar disorder [66]. We recognise the substantial contribution from these epidemiological studies and intend to incorporate the dimension of income inequality into our study explicitly.

### Institutional background

In Italy, mental health services are offered by the Italian National Health Service (INHS) through a network of community and hospital services. Access is completely free for hospital care, while outpatient specialist services require co-payment. Moreover, broad categories of patients are exempted from such co-payment for economic reasons (low income), age (elderly) or due to specific chronic conditions. With the approval of the Psychiatric Reform in 1978, new admissions to specialised mental institutions were banned (with the exclusion of forensic detention centres), psychiatric hospitals were gradually closed down, and acute hospital care was attributed entirely to general hospitals [67]. As a general rule, psychiatric services are organised around a department in charge of acute hospital care, outpatient services, day-care activities, including psychological treatments, rehabilitation and social services [67]. Although the national legislation requires uniform standards across the country, significant inter- and even intra-regional differences persist after almost 40 years of policies towards geographical equity. In particular, southern regions tend to offer fewer services, mainly community based [68].

The crisis in 2008 hit Italy with some specificities. First, the country’s economic performance was stagnating since the early 1990s. The average real GDP growth in the periods of 1993–2008 and 2009–2018 were merely 0.7% and –0.3%, respectively [69]. The great crisis hit an economy that was already strained by weak demand, lack of private investment, high public debt and declining international competitiveness in major industrial sectors. Moreover, government policies in Italy are constrained severely by its high public debt, so any attempt to use Keynesian policies to stimulate the economy with higher public spending is limited by tight budget constraints and the Euro Zone rules.

Unemployment rates have been persistently high since the onset of the crisis, especially among younger adults. In 2018, the employment rate for the population aged between 18 and 64 was 58.5%, almost 10% lower than that the average level registered for EU 28 countries [70]. Given the social structure and the conditions of the labour market, the employment rate is particularly low among the youth—with 43.4%, Italy breaks the EU record for being the country with the lowest employment rate for the age group of 20–29 [70]. Mean values for the leading indicators of economic performance mask significant geographical variations with some areas of the South being one of the poorest and most disadvantaged among all European countries. Southern regions, comprising about one-third of the Italian population, register a GDP per inhabitant that is less than 50% of Lombardy, the wealthiest region of the north [70]. Overall, the impact of the crisis primarily exhibits in the form of rising unemployment.

### Objectives

Using a societal perspective, we aim to carry forward the discussion by establishing the causality of deteriorating economic conditions during the economic crisis on mental disorder admissions in the Italian context. The study’s objectives are twofold: (i) to test and measure the causal impact of the economic crisis on mental disorder admissions in Italy; (ii) to assess the heterogeneous impact of the crisis across areas with distinct levels of income. We wish to not only provide evidence on the socioeconomic determinants of mental health admissions but also potentially connect the research to the policy debates on mental health and health care.

## Methods

### Data

We use administrative data from three primary sources and utilise the small geographic level as the unit of analysis to construct a panel data structure. First, we use the hospital discharge dataset collected by the Italian National Ministry of Health on all inpatient admissions during the period 2007–2015. The hospital discharge data provide detailed information about the clinical characteristics of the admitted patients, mainly through indications up to five secondary diagnoses. We requested for the extraction of patients aged between 18 and 65 and diagnosed with affective disorders (ICD-9: 296.0-296.9), which include severe mental disorders such as bipolar disorder, major depressive disorder and manic disorder. Our choice of age category is informed by our objective to detect the effect among individuals in the labour market, who tend to experience stress due to changing employment status and prospects. In investigating the socioeconomic determinants of mental disorders, many studies have focused specifically on affective disorders (or mood disorders), which is a subset of severe mental disorders including bipolar I disorder, major depressive disorder and manic disorder [10]. Patients with affective disorders face substantial morbidity and mortality, as well as social consequences, with life expectancy lower than average [71]. It is estimated that 7.4 % of the global disability-adjusted Life years (DALYs) are caused by diseases in the mental and behavioural disorder categories, with major depressive disorder carrying the most onerous burden. Therefore, we focus on this subgroup of affective disorder patients. Even though they represent only a limited fraction of all mental disorder categories, they have the highest admission volume in our dataset and are likely to be more associated with socioeconomic shocks rather than other mental diseases such as schizophrenia, which we consider for the placebo tests.

In the dataset, each patient is geographically located within one of the overall 611 Local Labour Areas—“Sistema Locale del Lavoro” (SLL)—that aggregates the neighbouring municipalities (“comuni”) to reflect a common economic structure [48]. The SLLs draw a territorial grid whose boundaries are drawn using the flows of daily work (commuting) detected from the general census of the population and households [72]. This local labour market system represents the ideal geographic unit of analysis, as individuals residing within the area by construction experience similar labour market changes due to the economic crisis. We, therefore, utilise the unemployment rate, labour market and population information at the SLL level obtained from the Italian National Statistical Office (ISTAT). For each SLL, changes in the annual unemployment rates are used as an indicator of crisis intensity in the labour market. Furthermore, since we exploit the variation in the unemployment rate across 9 years, we do not specify a restricted definition regarding the timing of economic crisis but rather regard it as a process. To control for the overall resources within the community, we further incorporated the dataset with the distribution of the population income and constructed residents’ stated income per person and the Gini coefficients at the SLL level.

Overall, we created a panel dataset of variables regarding patient admission, the unemployment rate, income level and other characteristics at the SLL level. The structure of the 9-year panel dataset with 611 areas per year allows us to identify the causal effect of unemployment change on mental disorder admission by eliminating the time-invariant unobserved heterogeneity.

### Econometric model

We exploit the nine years panel dataset and connect variations in admission for affective disorders per 100,000 residents to changes in unemployment rate across time and space. Panel datasets have some appealing characteristics: (1) it allows us to control for individual (for our purpose the SLL area) heterogeneity, (2) it gives more informative data—more variability, less collinearity among the variables, more degrees of freedom and more efficiency. To address the issue of potential omitted variable bias for unemployment rate on admission rate, we considered several identification strategies, and we explain each in turn briefly.

The most commonly used panel data model to eliminate unobserved effects is to apply the within (demeaning) transformation—the one-way fixed-effects (FE) model or to take first differences to exploit variation across periods. We tested the two models against pooled-OLS and random effect models and concluded that the FE estimator is consistent. We, therefore, consider the following equation:

1 $$\begin{aligned} \begin{array}{@{}l}{\text {adm}}_{it}=\beta \;{\text {Unemployment}}_{it}+{\varvec{X}}_{\mathbf{i}\mathbf{t}}\varvec{\;}\gamma +u_i+\;\varepsilon _{it},\end{array} \end{aligned}$$

where ${\rm{adm}_{it}}$ denotes the number of affective disorder admissions per 100,000 residents for the area *i *at year *t. *Variable ${\rm{unemployment}}_{it }$ is the unemployment rate for the area *i* at year *t*. The coefficient ${{\beta }}$ is of primary interest as it represents the impact of labour market condition on admissions for mental disorders. ${{\varvec{X}}}_{it}$ is a vector of control variables that include average income per capita, the Gini coefficient, family size, gender composition and other aggregated patient characteristics at the SLL level. ${u_{i }}$ is the unobserved area heterogeneity that is time invariant such as rurality or general population composition, while ${\varepsilon }_{it }$ is the idiosyncratic error term. We assume the error term ${\varepsilon }_{{it }}$ to be independent and identically distributed $\varepsilon _{it}\;\sim IID(0,\delta _\varepsilon ^{2})$ and ${{\varvec{X}}}_{it}$ to be independent of the ${\varepsilon }_{it }$ for all *i *and *t*. We estimate Eq. (1) using fixed-effect panel estimation, where we cluster robust standard errors at the SLL level and include a set of year dummies. We also estimate the same equation using time-lagged explanatory variable ${\rm{unemployment}}_{{i,t-1 }}$ to allow for delayed effects on mental disorder admission. We complement the fixed-effect model with the alternative first-difference estimation, where time-invariant area-specific effects are cancelled over time.

One can argue that the relationship between an increased unemployment rate and mental disorder hospitalisation involves an adjustment process. This dynamic happens when the year’s outcome depends not only on the independent variables but also on the outcome of the previous year. Moreover, standard linear panel models, despite their various merits, can suffer from biases due to short time duration. We, therefore, expand our analysis to a dynamic panel model that includes the lagged value of the dependent variable.

2 $$\begin{aligned} \begin{array}{@{}l}{\rm{adm}}_{it}=\alpha \;{\rm{adm}}_{i,t-1}+\beta \;{\rm{Unemployment}}_{it}+{\varvec{X}}_{\mathbf{i}\mathbf{t}}\,\gamma +u_i+\varepsilon _{it}\end{array} \end{aligned}$$

where $u_i\;\sim \;IID(0,\;\delta _u^{2})$ and $\varepsilon _{ti}\;\sim \;{\rm{IID}}(0,\;\delta _\varepsilon ^{2})$ are assumed to be independent of each other and among themselves. The dynamic panel regression has two sources of persistence over time—autocorrelation due to a lagged dependent variable and area heterogeneous effects [73]. Within-group estimators for the above equation can result in bias as elimination of $\textit{u}_{i}$ can cause correlations between the transformed error term and the transformed lagged dependent variable. We, therefore, also perform first difference transformation and allow the use of lags of ${\rm{adm}}_{{i,t \, -1}}$ as valid instruments [73–75].

First difference with lagged adm:

3 $$\begin{aligned} \begin{array}{@{}l}\triangle {\rm{adm}}_{it}=\alpha \triangle {\rm{adm}}_{i,t-1}+\beta \triangle {\rm{unemployment}}_{it}+\triangle {\varvec{X}}_{\mathbf{i}\mathbf{t}}\gamma +\triangle \;\varepsilon _{it},\\t\;=\;2,\;...T\end{array} \end{aligned}$$

Since $\triangle {\rm{admi}}_{i,t-2}$ is clearly correlated with $\triangle {\rm{admi}}_{i,t-1}={\rm{adm}}_{i,t-1}-adm_{i,t-2}$ but not with the error term $\triangle \varepsilon _{it}=\varepsilon _{it}-\varepsilon _{i,t-1}$ , it can be a valid instrument. We estimated the equation using both Anderson and Hsiao estimator [76] and Arellano and Bond estimator [74] based on Generalised Methods of Moments (GMM). For the former, we instrument the lagged dependent variable with twice-lagged level, while for the latter model we combine the first differences with a model using lagged differences as instruments [77]. Lags of ${\rm{unemployment}}_{i,t-1}$ are also used as an instrument for unemployment. We include a full set of year dummies.

While we aim to capture the causal impact of changing unemployment on mental disorder admissions, we are also interested in the heterogeneous effects of the crisis across socioeconomic groups. In the last section of the analysis, we create discrete quintile groups according to the area’s income level and interact the groups with the unemployment rate for the panel fixed-effect model.

4 $$\begin{aligned} \begin{array}{@{}l}\begin{array}{l}{\rm{adm}}_{it}=\beta \;{\rm{unemployment}}_{it}\;+\delta ({\rm{unemployment}}_{it}\cdot {\rm{Income\_quintile}}_{it})\;\;\\ +\eta \;{\rm{Income\_quintile}}_{it}+{\varvec{X}}_{\mathbf{i}\mathbf{t}}\;\gamma +u_i+\varepsilon _{it}\end{array}\end{array} \end{aligned}$$

The parameter ${{\delta }}$ identifies the differential effect of the interaction term between the indicator of the area belonging to one of the income level quintiles and the unemployment rate. The coefficients for the different quintile levels represent the crisis effect on admission rate for that income quintile area, and we use both the fixed-effect and dynamic within transformation to estimate the heterogeneous impact.

## Results

### Descriptive statistics

We have a balanced panel of 9 years and 611 SLLs. The summary of the variables we constructed can be found in Table 1. We observe that overall, the admission rate for all affective disorder hospitalisation is around 77 individuals per 100,000 population on average for the SLLs, with the majority being bipolar disorder admissions. The average age of the patients is around 43 years, and the average length of stay is around 13 days. The unemployment rate is about 10% but ranges from 1.42 to 38.7%, indicating a considerable variation across areas. Income and inequality measures also differ widely across areas.

**Table 1:** Descriptive statistics

Variables (average at SLL level)	Values			
	Mean	S.D.	Min	Max
Admissions for all affective disorder/100,000	77.164	47.483	0	384.97
Admissions for bipolar I disorder/100,000	36.863	28.005	0	259.99
Admissions for major depressive disorder/100,000	21.511	22.539	0	261.51
Admissions for manic disorder/100,000	1.073	3.108	0	131.30
Patient age	43.215	2.278	27.75	57.71
Length-of-stay	12.564	4.577	2	66.87
Unemployment rate (%)	10.246	5.597	1.42	38.70
Annual declared income per person	11,509	3,084	5,077	20,949
Population of residents	97,208	257,904	3,156	3,682,555
Gini coefficient (*100)	14.106	1.821	8.67	24.67
Family size	2.390	0.220	1.55	3.36
Proportion of male (%)	48.78	0.77	46.267	53.834
Total observations	5,499			

**Fig. 1:**
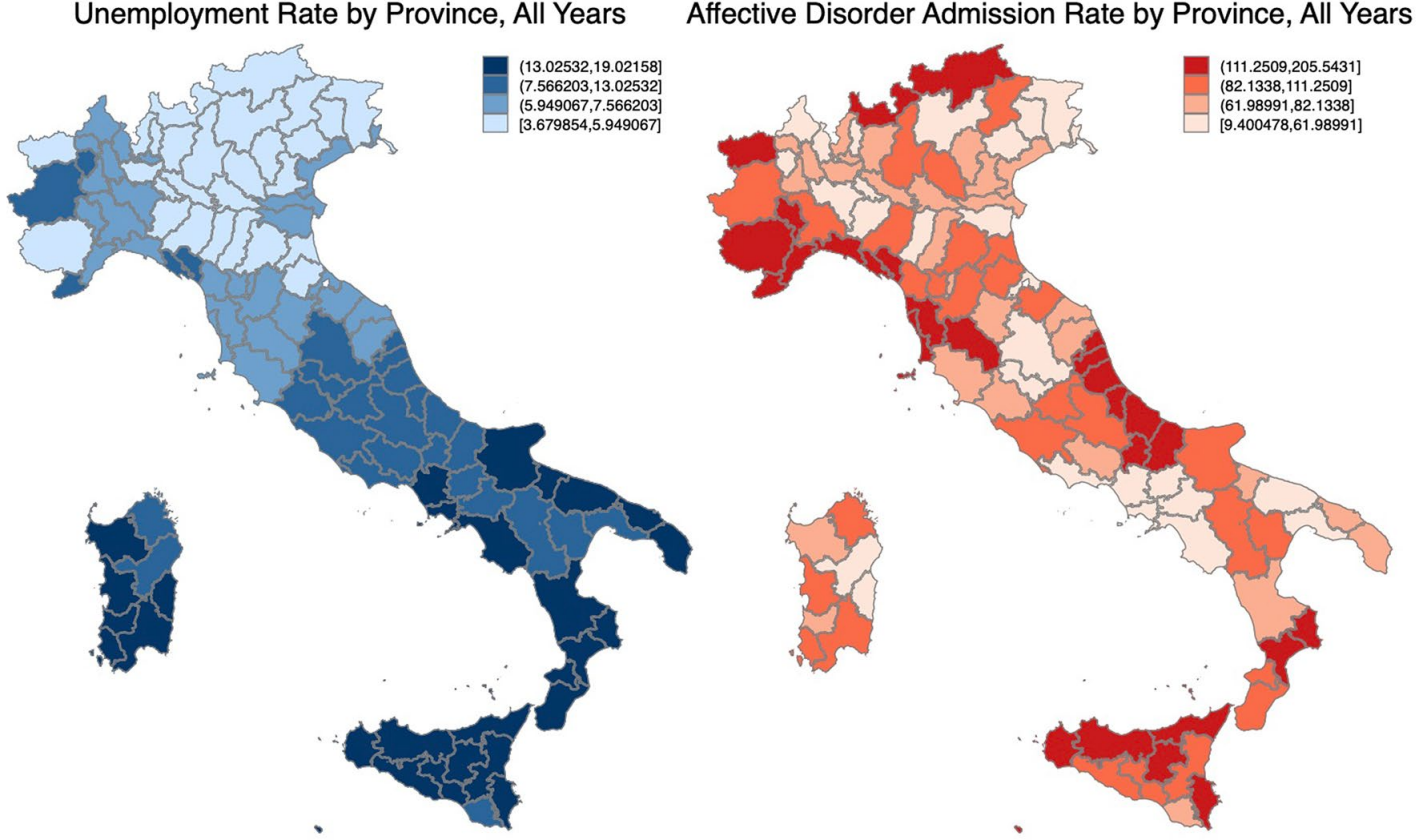
Geographic distribution of unemployment rate and affective disorder admissions, all years

**Fig. 2:**
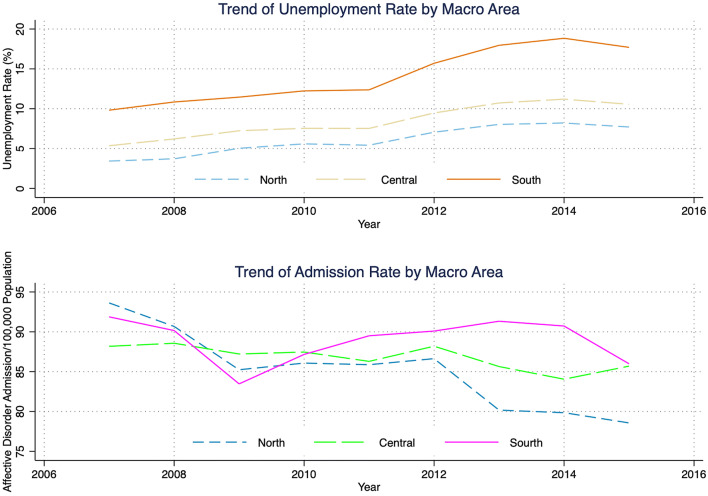
Time trends of unemployment rate and affective disorder admission rate, by macro area

We further characterise the variation of our variables of interest over geography and time. The spatial variation of the average unemployment rate is found in Fig. 1 on the left, where we observe a visible gradient between the north and the south. However, affective disorder admissions do not appear to have a clear geographic pattern. Over time, we see in Fig. 2 that the unemployment rate increases consistently since 2008 and peaked in 2014, with the south having persistently higher levels than the central and northern regions. While total hospitalisation per 100,000 residents initially declined until 2009, it then experienced a drastic increase in the south. Descriptively, it appears that the increase in the unemployment rate over the observed period is accompanied by an increase in the admission for affective disorder patients for the southern regions, while it is ambiguous for the central and northern regions. What we aim to capture is the (heterogeneous) effects of the worsening labour market conditions on admissions for affective disorder.

**Fig. 3:**
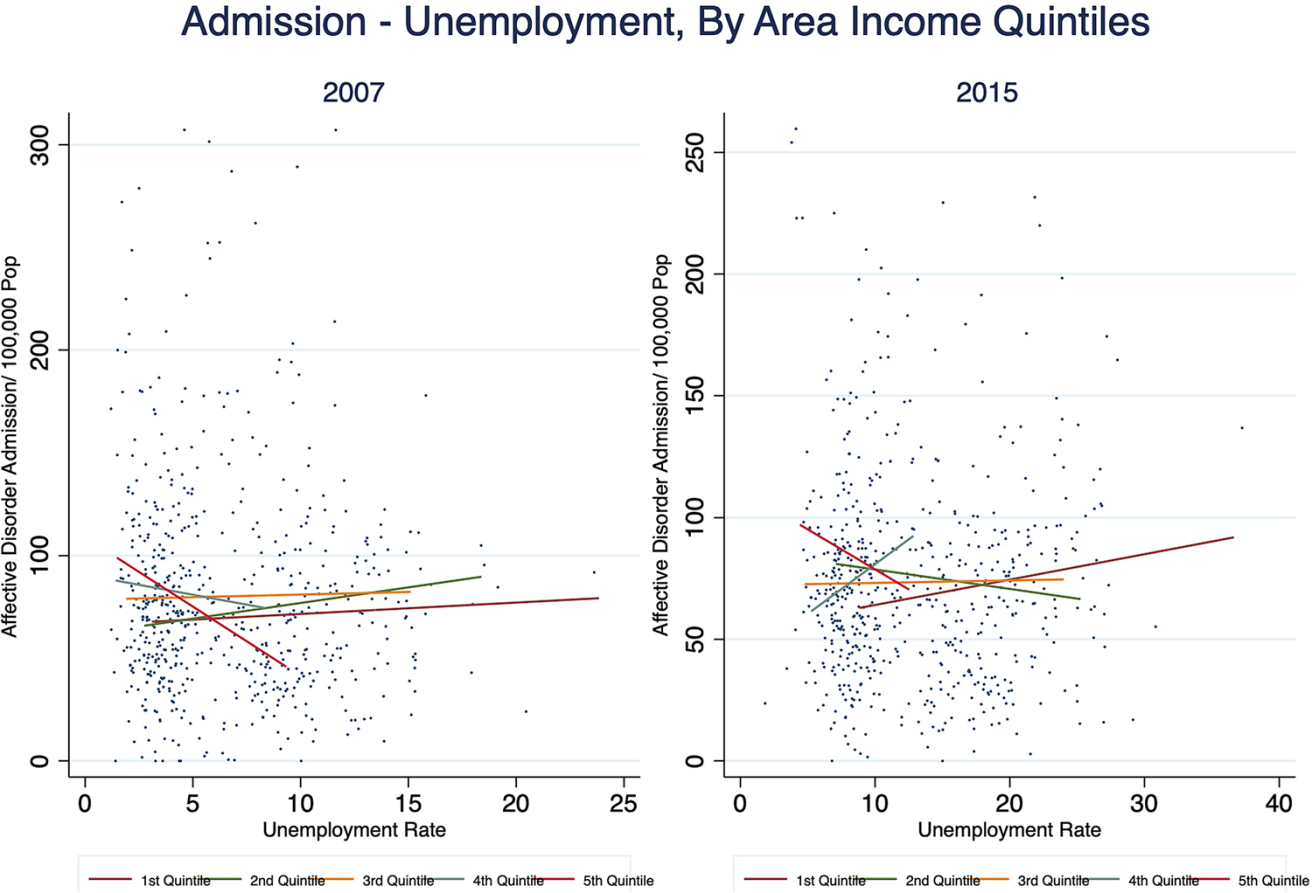
Scatterplot of admission rate against unemployment rate by area income quintiles, 2007 & 2015

In understanding the socioeconomic gradient of the correlation, we plotted the two variables of interest across discrete quintile groups according to the average declared income per person (Fig. 3). The scatterplot shows that in 2007 the correlation is slightly negative with no substantial differences across the quintile groups; but by 2015, there is a positive correlation between unemployment rates and admission rates for the first and the fourth income quintile groups (the dark red and the dark green lines). It means that descriptively, the effect of the crisis on mental disorder admissions differ across areas characterised by distinct economic conditions.

### Regression results

Table 2 reports the fixed-effect and first difference estimators from Eq. (1). For all the models, there are significant and positive effects of unemployment rate on the admission rate for affective disorder—1 percentage point increase in unemployment gives rise to about 1 out of 100,000 residents being admitted to the hospital due to affective disorder. Although the result is robust for unemployment, most of the control variables are not significant.

**Table 2:** Linear panel models

Models	FE	FE with lagged unemployment	FD
Variables	Admission	Admission	Admission
Unemp	1.618** *		0.849**
	(0.407)		(0.331)
Lagged Unemp		1.052**	
		(0.415)	
Income per capita	0.000616	0.000303	–0.000338
	(0.00103)	(0.00111)	(0.00101)
Gini (*100)	0.432	–0.107	–0.209
	(1.108)	(1.175)	(0.970)
Patient age	0.0273	0.235	0.174
	(0.290)	(0.300)	(0.196)
Length-of-stay	0.0342	–0.204	0.158
	(0.234)	(0.242)	(0.151)
Family size	11.38	15.45	–5.774
	(10.95)	(11.33)	(9.670)
Proportion of male (%)	–1.302	–1.055	–4.068**
	(1.576)	(1.624)	(1.728)
Constant	89.74	75.69	–1.384**
	(93.81)	(96.17)	(0.584)
Observations	5499	4888	4888
Year dummy	Included	Included	
*F*-statistics	2.99***	2.43***	2.35***
Number of small areas	611	611	

Standard errors in parentheses

***p<0.01, **p<0.05, *p<0.1

For the dynamic panel models, we report in Table 3 the fixed effect estimator with lagged dependent variable from Eq. (2), the Anderson and Hsiao estimator as well as the Arellano and Bond estimator from Eq. (3). Consistent with the linear panel model, all the coefficients for unemployment in the dynamic panel models are positive and significant, with values around 1. The similar results across linear and dynamic panel models show strong evidence for the effect of unemployment on admissions for affective disorder. The specification test for GMM shows that there is first-order serial correlation (AR1), and no second-order serial correlation (AR2). The Hansen test does not reject the over-identifying conditions.

**Table 3:** Dynamic panel model

Models	Within transformation	Anderson and Hsiao	Arellano and Bond
Variables	Admission	Admission	Admission
Lagged Adm	0.149***	0.274***	0.0927
	(0.0359)	(0.0517)	(0.0629)
Unemp	1.017***	0.679*	1.460*
	(0.315)	(0.384)	(0.770)
Income per person	0.000730	–0.000410	–0.00378
	(0.00102)	(0.00122)	(0.0125)
Gini (*100)	–0.196	–0.611	–7.267
	(1.077)	(1.136)	(7.407)
Patient age	0.240	0.352	3.255
	(0.299)	(0.235)	(5.840)
Length-of-stay	–0.123	0.139	7.936**
	(0.234)	(0.190)	(4.021)
Family size	5.901	–6.396	–134.2*
	(9.579)	(11.37)	(69.38)
Proportion of male (%)	–1.995	–4.681**	13.26
	(1.474)	(2.004)	(22.63)
Constant	–1.121***	–1.036	–5.890**
	(0.396)	(0.698)	(2.600)
Year dummy	Included	Included	Included
Observations	4888	4277	4277
Number of small areas	611		611
Instruments		7	29
Hansen test Chi square			27.04
AR1 test			–3.91***
AR2 test			2.10**
*F* statistic	5.81***		3.26 ***

Standard errors in parentheses

***p<0.01, **p<0.05, *p<0.1

To check the robustness of our findings, we run both the linear and dynamic panel model for the sub-categories of affective disorders— bipolar disorder and major depressive disorder. For each disorder, we first use the fixed-effect estimator with and without the 1-year lag of unemployment rate, as well as the Arellano–Bond estimator with a 1-year lag. As seen in Table 4, the impact of unemployment is significant and robust for major depressive disorder admissions across all models, and similar for bipolar disorder except for the FE estimator with the lagged unemployment rate.

**Table 4:** Sub-disorder admissions

Models	FE	FE	FE lagged	FE lagged	Arellano-Bond	Arellano–Bond
	Bipolar I	Depressive	Bipolar I	Depressive	Bipolar I	Depressive
Lagged Adm					0.0557	0.0372
					(0.0355)	(0.0313)
Unemp	0.597***	0.889***			0.734**	0.675**
	(0.215)	(0.257)			(0.308)	(0.282)
Lagged Unemp			0.0875	0.498***		
			(0.177)	(0.144)		
Income per capita	0.000620	0.000740	0.000799	0.000219	0.000454	-0.00285
	(0.000727)	(0.000619)	(0.000612)	(0.000500)	(0.00493)	(0.00400)
Gini (*100)	0.473	–0.561	–0.364	-0.400	–1.306	–0.669
	(0.755)	(0.690)	(0.611)	(0.499)	(2.910)	(2.408)
Patient age	0.151	0.339**	0.301**	0.309***	0.200	4.967
	(0.188)	(0.158)	(0.146)	(0.119)	(2.263)	(4.558)
Length-of-stay	–0.0161	0.145	–0.127	0.0531	2.307*	2.618
	(0.129)	(0.116)	(0.107)	(0.0875)	(1.377)	(2.434)
Family size	10.92	–4.356	1.966	-2.800	—55.04	–43.70
	(6.827)	(6.197)	(5.273)	(4.304)	(35.22)	(34.31)
Male (%)	–0.543	0.0971	–0.990	-0.439	–4.449	7.602
	(0.999)	(0.926)	(0.946)	(0.772)	(12.47)	(10.94)
Constant	11.52	8.771	–478.4	2,539***		
	(56.87)	(52.05)	(487.7)	(398.0)		
Observation	5499	5499	4888	4888	4277	477
Year dummy	Included	Included	Included	Included	Included	Included
Instruments					30	30
Hansen test Chi square					30.31*	22.73
AR1 test					0.049	–7.24***
AR2 test					0.562	–2/09**
*F* statistic	3.33***	5.74***	3.09***	8.55***	5.64***	2.59***

Standard errors in parentheses

***p<0.01, **p<0.05, *p<0.1

In identifying the potential gradients of the crisis effect on admission rate, we interact the different income quintiles with the unemployment rate as indicated in Eq. (4). We observe in Table 5 that for both the linear and dynamic panel models, the impact of unemployment is only significant for areas belonging to the 1st quintile of average income (the coefficient for Unemployment), and, curiously, the marginal effects are negative for areas belonging to the top quintiles of average income. We can reasonably conclude that the adverse impact of rising unemployment admission for affective disorders is concentrated on the most economically disadvantaged areas.

**Table 5:** Heterogeneous effects across area income quintiles

odels	Fixed effect	Dynamic within
Variables	Admission	Admission
Lagged adm		0.148***
		(0.0356)
Unemp	0.661**	0.506*
	(0.304)	(0.286)
2 Quintile Inc	–3.305	–2.032
	(7.406)	(7.425)
3 Quintile Inc	14.69*	14.33*
	(8.885)	(8.542)
4 Quintile Inc	10.24	6.813
	(8.885)	(8.678)
5 Quintile Inc	18.51**	14.64
	(9.090)	(9.010)
2 Quintile Inc * Unemp	0.393	0.425
	(0.405)	(0.380)
3 Quintile Inc * Unemp	-0.790*	–0.683
	(0.465)	(0.424)
4 Quintile Inc * Unemp	–0.551	0.116
	(0.634)	(0.605)
5 Quintile Inc * Unemp	–1.768***	–1.082*
	(0.596)	(0.579)
Gini coefficient (*100)	1.302	0.244
	(1.036)	(1.038)
Patient age	-0.0126	0.187
	(0.287)	(0.298)
Length-of-stay	–0.00637	–0.177
	(0.238)	(0.238)
Family size	16.28*	14.56*
	(8.341)	(8.321)
Proportion of male (%)	–2.815**	–2.843**
	(1.366)	(1.312)
Constant	146.7*	149.8**
	(75.03)	(72.21)
Observations	5499	4888

Standard errors in parentheses

***p<0.01, **p<0.05, *p<0.1

### Placebo test

We further run a placebo test for the hospitalisation rate of schizophrenic patients. There is ample evidence in social psychiatry research that schizophrenia is not associated with sudden labour market changes but with urbanicity and socio-environmental changes, usually with an early onset during teenage years [78, 79]. Although we believe that increasing social fragmentation and income decline may contribute to early onsets of schizophrenic patients, job loss and labour market deterioration should not affect the hospitalisation of these patients. Indeed, in our placebo regression for admission rates of schizophrenic patients, we observe an insignificant effect of the unemployment rate in all specifications (Table 6). Whereas for the Gini coefficient, the proxy for income inequality, significantly contributes to the admissions for schizophrenia. Further research is warranted to investigate the mechanism behind rising societal inequality during economic downturns and the likely onset of schizophrenia symptoms.

**Table 6:** Placebo regression for schizophrenia admission

Models	FE	FE with lagged unemployment rate	Arellano-Bond
Lagged Adm			0.0523
			(0.235)
Unemp	–0.251		0.466
	(0.410)		(0.570)
Lagged Unemp		–0.0231	
		(0.403)	
Income per capita	–0.000401	–4.97e–05	0.000763
	(0.000784)	(0.000795)	(0.00845)
Gini (*100)	2.672**	2.517**	1.480
	(1.048)	(1.018)	(4.634)
Patient age	-0.382	-0.302	–1.937
	(0.240)	(0.264)	(2.876)
Length-of-stay	–0.510***	-0.688**	–2.145
	(0.183)	(0.277)	(2.304)
Family size	–22.31**	–15.05*	–0.532
	(9.903)	(9.028)	(46.23)
Proportion of male (%)	-1.236	–0.273	1.071
	(1.339)	(1.229)	(14.27)
Constant	168.0**	96.45	
	(78.07)	(72.05)	
Observation	5499	4888	4277
Year dummy	Included	Included	Included
Instruments			29
Hansen test Chi square			14.88
AR1 test			–1.35
AR2 test			–0.93
*F* statistic	16.44***	12.49***	12.77***

Standard errors in parentheses

***p<0.01, **p<0.05, *p<0.1

## Discussion

Our analysis has shown strong evidence for the impact of the economic crisis on admissions for affective disorders for the entire population in Italy. The effect is significant for all the different models that we tested, even though the magnitude is moderate. We argue that since we observe only inpatient admissions, not outpatient interventions, the actual impact could be even more severe. Moreover, it is well established that affective disorders are associated to cardiovascular diseases [80], and thus the impact of unemployment on wellbeing and health care utilisation is likely to be substantially higher than that measured in this study. We also recognise that our outcome variable is limited in the sense that admission per se is a combination of supply and demand factors. While increasing inpatient admissions could reflect a greater need for care, it could be a result of a lack of ambulatory care and consequently use of hospital care when it is not appropriate. If this is the case, it will change partly our interpretations of the result, but we are nonetheless capturing the impact of rising unemployment on mental health care utilisation. Moreover, since the inpatient hospitalisation for affective disorder covers severely ill patients and not patients seeking counselling or outpatient visits, we believe the supply-side influence on admission is minimal. Finally, our results could be subject to migration bias, as individuals may move from more deprived areas to more affluent areas during the crisis. We have qualitatively assessed this possibility and did not observe a systematic change in the resident population over the years. In our dataset, we observe only around 8% of the patients seeking care in a region outside of his/her residence, indicating a low likelihood of patients travelling. Nonetheless, the exact pattern of population mobility is beyond our capacity to investigate given the nature of our data.

Our study uniquely contributes to the stream of literature on the socioeconomic determinants of mental illness by establishing the causal impact of rising unemployment during the economic crisis on severe mental disorder admissions in the context of universal coverage. The linear and dynamic panel models that we tested all point to the same conclusion—higher unemployment increases admission for affective disorder. However, inequality did not play a contributing role. When we analyse the socioeconomic gradient of the impact, we have found that areas with the lowest levels of income per capita are the most affected population. The result shows how people who belong to the more economically vulnerable segment of the society can experience adverse episodes due to their mental distress towards the deteriorating economic environment. Behind this effect, two mechanisms may be at play: (i) for the unemployed, worsening labour market conditions could have induced the onsets of affective disorders; (ii) for the employed, the social diffusion of job insecurity has raised the anxiety level that potentially led to affective disorder. The findings are in line with the materialist explanation for psychological stress, as adverse economic conditions may have contributed to the chronic arousal of the stress system for those who are either unemployed or live in a neighbourhood that is profoundly affected by unemployment.

The recent COVID-19 outbreak has brought another heavy storm to harm the mental health of the population in Italy. With increased social isolation, the general sense of grief and fear, alongside the grim economic prospects, we can reasonably expect anxiety, stress, and potential mental illness to escalate. Moreover, individuals who have existing mental health conditions may face challenges in their access to care and service continuity due to the interruptions in the health care system. The findings of our research can be critically relevant for the socioeconomic crisis that follows the pandemic disaster. We, therefore, hope to pave the way for more empirical evidence on the ramification of the COVID-19 crisis on the mental health care in various countries.

An estimated 970 million people around the world suffer from mental distress, and the prevalence of, for instance, depression has risen more than 40% over the past 30 years [71]. The overwhelming phenomenon reflects a combination of the rising needs and the increasing awareness to seek treatment. How society perceives mental illness patients and how health care systems allocate resources to treatment and social policies will be a long-lasting debate. We hope that our study can bring to light the importance of adequate policy responses to address the psychological aspects of large-scale socioeconomic shocks in the long term. Specifically, more resources should be invested in social services and mental health specialist, both at the workplace and at the community level, to meet the future surge of needs and to prevent the loss of human capital and consequently labour market opportunities.

## References

[1] Faris REL, Dunham HW (1939). Mental disorders in urban areas: an ecological study of schizophrenia and other psychoses.

[2] Kammerling RM, O’Connor S (1993). Unemployment rate as predictor of rate of psychiatric admission. BMJ Br Med J.

[3] Goldsmith AH, Veum JR, William D (1996). The impact of labor force history on self-esteem and its component parts, anxiety, alienation and depression. J Econ Psychol.

[4] Reijneveld SA, Schene AH (1998). Higher prevalence of mental disorders in socioeconomically deprived urban areas in The Netherlands: Community or personal disadvantage. J Epidemiol Commun Health.

[5] Ross CE, Reynolds JR, Geis KJ (2000). The contingent meaning of neighborhood stability for residents. psychological well-being. Am Sociol Rev.

[6] Silver E, Mulvey EP, Swanson JW (1982). Neighborhood structural characteristics and mental disorder: Faris and Dunham revisited. Soc Sci Med.

[7] Gili M, Roca M, Basu S, McKee M, Stuckler D (2013). The mental health risks of economic crisis in Spain: evidence from primary care centres, 2006 and 2010. Eur J Public Health.

[8] Urbanos-Garrido RM, Lopez-Valcarcel BG (2015). The influence of the economic crisis on the association between unemployment and health: an empirical analysis for Spain. The European Journal of Health Economics: HEPAC: Health Economics in Prevention and Care.

[9] Madianos M, Economou M, Alexiou T, Stefanis C (2011). Depression and economic hardship across Greece in 2008 and 2009: two cross-sectional surveys nationwide. Soc Psychiatry Psychiatr Epidemiol.

[10] Economou M, Madianos M, Peppou LE, Patelakis A, Stefanis CN (2013). Major depression in the era of economic crisis: a replication of a cross-sectional study across Greece. J Affect Disord.

[11] Modrek S, Hamad R, Cullen MR (2015). Psychological well-being during the great recession: changes in mental health care utilization in an occupational cohort. Am J Public Health.

[12] Thern E, Munter JD, Hemmingsson T, Rasmussen F (2017). Long-term effects of youth unemployment on mental health: Does an economic crisis make a difference. J Epidemiol Commun Health.

[13] Barr B, Kinderman P, Whitehead M (2015). Trends in mental health inequalities in England during a period of recession, austerity and welfare reform. Soc Sci Med.

[14] Buffel V, van de Straat V, Bracke P (2015). Employment status and mental health care use in times of economic contraction: a repeated cross-sectional study in Europe, using a three-level model. Int J Equity Health.

[15] Curtis S, Pearce J, Cherrie M, Dibben C, Cunningham N, Bambra C (2019). Changing labour market conditions during the ‘great recession’ and mental health in Scotland 2007–2011: An example using the Scottish Longitudinal Study and data for local areas in Scotland. Soc Sci Med.

[16] Drydakis N (2015). The effect of unemployment on self-reported health and mental health in Greece from 2008 to 2013: a longitudinal study before and during the financial crisis. Soc Sci Med.

[17] Schaller J, Stevens AH (2015). Short-run effects of job loss on health conditions, health insurance, and health care utilization. J Health Econ.

[18] Bobes J, García CI, González MPG-P, Bascarán MT, Treviño LJ, Pelayo-Terán JM, Revuelta JR, Lasheras FS, Martínez PS (2013). Changes in administrative prevalence of mental disorders over a 13-year period in Asturias (northern Spain). Revista De Psiquiatria Y Salud Mental.

[19] García CI, Martinez PS, González MPG-P, García MB, Treviño LJ, Lasheras FS, Bobes J (2014). Effects of the economic crisis on demand due to mental disorders in Asturias: data from the Asturias Cumulative Psychiatric Case Register (2000–2010). Actas Espanolas De Psiquiatria.

[20] Lagravinese R (2015). Economic crisis and rising gaps North–South: evidence from the Italian regions. Camb J Regions Econ Soc.

[21] Belvis AGD, Ferrè F, Specchia ML, Valerio L, Fattore G, Ricciardi W (2012). The financial crisis in Italy: Implications for the healthcare sector. Health Policy.

[22] Minelli L, Pigini C, Chiavarini M, Bartolucci F (2014). Employment status and perceived health condition: longitudinal data from Italy. BMC Public Health.

[23] Vogli RD, Vieno A, Lenzi M (2014). Mortality due to mental and behavioral disorders associated with the Great Recession (2008–10) in Italy: a time trend analysis. Eur J Public Health.

[24] Elstad JI (1998). The psycho-social perspective on social inequalities in health. Sociol Health Illn.

[25] Schnall PL, Landsbergis PA, Baker D (1994). Job strain and cardiovascular disease. Annu Rev Public Health.

[26] Bosma H, Marmot MG, Hemingway H, Nicholson AC, Brunner E, Stansfeld SA (1997). Low job control and risk of coronary heart disease in Whitehall II (prospective cohort) study. BMJ.

[27] Bosma H, Peter R, Siegrist J, Marmot M (1998). Two alternative job stress models and the risk of coronary heart disease. Am J Public Health.

[28] Landsbergis PA, Grzywacz JG, LaMontagne AD (2014). Work organization, job insecurity, and occupational health disparities. Am J Ind Med.

[29] Siegrist J, Peter R, Cremer P, Seidel D (1997). Chronic work stress is associated with atherogenic lipids and elevated fibrinogen in middle-aged men. J Intern Med.

[30] Blane, D.: Tackling Inequalities in Health: an agenda for action. Sociology of Health & Illness 19, no. 1, pp 127–128 (1997)

[31] Weber, M.: “Wirtschaft Und Gesellschaft: Grundriss Der Verstehenden Soziologie,” (1972/1922)

[32] Bartley, M.: Health inequality: an introduction to concepts, theories and methods. Wiley (2016)

[33] Wilkinson R (1999). Health, hierarchy, and social anxiety. Ann N Y Acad Sci.

[34] Latkin CA, Curry AD (2003). Stressful neighborhoods and depression: a prospective study of the impact of neighborhood disorder. J Health Soc Behav.

[35] Pearlin LI (1989). The sociological study of stress. J Health Soc Behav.

[36] Mirowsky, J., Ross, C.: Social causes of psychological distress. Walter De Gruyter Incorporated (1989)

[37] Marmot M (2004). Dignity and inequality. Lancet.

[38] Whitehead M, Pennington A, Orton L, Nayak S, Petticrew M, Sowden A, White M (2016). How could differences in ‘control over destiny’ lead to socio-economic inequalities in health? A synthesis of theories and pathways in the living environment. Health Place.

[39] McKee, M.: The health consequences of the collapse of the Soviet Union. Poverty, inequality, and health: an international perspective. Oxford University Press (2000)

[40] Fisher M, Baum F (2010). The social determinants of mental health: implications for research and health promotion. Aust N Zeal J Psychiatry.

[41] Kirkbride JB, Fearon P, Morgan C, Dazzan P, Morgan K, Murray RM, Jones PB (2007). Neighbourhood variation in the incidence of psychotic disorders in Southeast London. Soc Psychiatry Psychiatr Epidemiol.

[42] Reijneveld SA, Schene AH (1998). Higher prevalence of mental disorders in socioeconomically deprived urban areas in The Netherlands: community or personal disadvantage?. J Epidemiol Commun Health.

[43] Thornicroft G (1991). Social deprivation and rates of treated mental disorder. Developing statistical models to predict psychiatric service utilisation. Br J Psychiatry J Mental Sci.

[44] Weyerer S, Häfner H (1992). The high incidence of psychiatrically treated disorders in the inner city of Mannheim. Soc Psychiatry Psychiatr Epidemiol.

[45] Coveney M, García-Gómez P, Doorslaer EV, Ourti TV (2016). Health disparities by income in Spain before and after the economic crisis. Health Econ.

[46] Fichera E, Gathergood J (2016). Do wealth shocks affect health? New evidence from the housing boom. Health Econ.

[47] Ruhm CJ (2000). Are recessions good for your health?. Q J Econ.

[48] Torbica A, Maggioni AP, Ghislandi S (2015). The economic crisis and acute myocardial infarction: new evidence using hospital-level data. PLOS ONE.

[49] Ruhm CJ (2016). Health effects of economic crises. Health Econ.

[50] Belloni M, Meschi E, Pasini G (2016). The effect on mental health of retiring during the economic crisis. Health Econ.

[51] McInerney M, Mellor JM (2012). Recessions and seniors’ health, health behaviors, and healthcare use: analysis of the medicare current beneficiary survey. J Health Econ.

[52] Frasquilho D, Matos MG, Salonna F, Guerreiro D, Storti CC, Gaspar T, de Almeida JMC (2016). Mental health outcomes in times of economic recession: a systematic literature review. BMC Public Health.

[53] Parmar D, Stavropoulou C, Ioannidis JP (2016). Health outcomes during the 2008 financial crisis in Europe: systematic literature review. BMJ.

[54] Silva M, Resurrección DM, Antunes A, Frasquilho D, Cardoso G (2018). Impact of economic crises on mental health care: a systematic review. Epidemiol Psychiatr Sci.

[55] Bartoll X, Palència L, Malmusi D, Suhrcke M, Borrell C (2014). The evolution of mental health in Spain during the economic crisis. Eur J Public Health.

[56] Rivera B, Casal B, Currais L (2017). Crisis, suicide and labour productivity losses in Spain. Eur J Health Econ.

[57] Isabel R, Miguel R, Antonio R (2017). Economic crisis and suicides in Spain. Socio-demographic and regional variability. Eur J Health Econ.

[58] Mattei G, Ferrari S, Pingani L, Rigatelli M (2014). Short-term effects of the 2008 Great Recession on the health of the Italian population: an ecological study. Soc Psychiatry Psychiatr Epidemiol.

[59] Pompili M, Vichi M, Innamorati M, Lester D, Yang B, Leo DD, Girardi P (2014). Suicide in Italy during a time of economic recession: some recent data related to age and gender based on a nationwide register study. Health Soc Care Commun.

[60] Mattei G, Pistoresi B (2019). Eur J Health Econ.

[61] Lee CB, Liao C-M, Lin C-M (2017). The impacts of the global financial crisis on hospitalizations due to depressive illnesses in Taiwan: A prospective nationwide population-based study. J Affect Disord.

[62] Hsieh C-C, Pugh MD (1993). Poverty, Income Inequality, and Violent Crime: A Meta-Analysis of Recent Aggregate Data Studies. Crim Justice Rev.

[63] Rodgers GB (1979). Income and inequality as determinants of mortality: An international cross-section analysis. Popul Stud.

[64] Wilkinson RG, Pickett KE (2006). Income inequality and population health: a review and explanation of the evidence. Soc Sci Med.

[65] Ribeiro W S, Bauer A, Andrade M C R, York-Smith M, Pan P M, Pingani L, Evans-Lacko S (2017). Income inequality and mental illness-related morbidity and resilience: A systematic review and meta-analysis. Lancet Psychiatry.

[66] Wilkinson, R., Pickett, K.: The inner level: how more equal societies reduce stress, restore sanity and improve everyone's well-being. Penguin Book (2020) 10.3399/bjgp19X705377 PMC6715479 31467020

[67] Fattore G, Percudani M, Pugnoli C, Contini A, Beecham J (2000). Mental health care in Italy: organisational structure, routine clinical activity and costs of a community psychiatric service in Lombardy Region. Int J Soc Psychiatry.

[68] Bollini P, Reich M, Muscettola G (1988). Revision of the Italian psychiatric reform: north/south differences and future strategies. Soc Sci Med.

[69] OECD.: OECD data (2018). [Online]. Available: https://data.oecd.org/

[70] Eurostat.: Eurostat 2019. [Online]. https://ec.europa.eu/eurostat/home

[71] WHO.: World Health Organization | Mental Health 2018. [Online]. https://www.who.int/news-room/fact-sheets/detail/mental-disorders

[72] ISTAT.: (2011). [Online]. https://www.istat.it/it/informazioni-territoriali-e-cartografiche/sistemi-locali-del-lavoro

[73] Baltagi B H (2005). Econometric Analysis of Panel Data.

[74] Arellano M, Bond S (1991). Some tests of specification for panel data: Monte Carlo evidence and an application to employment equations. Rev Econ Stud.

[75] Arellano M, Bover O (1995). Another look at the instrumental variable estimation of error-components models. J Econ.

[76] Anderson T, Hsiao C (1981). Estimation of Dynamic models with error components. J Am Statist Assoc.

[77] Blundell R, Bond S (1998). Initial conditions and moment restrictions in dynamic panel data models. J Econ.

[78] Silver E, Mulvey EP, Swanson JW (2002). Neighborhood structural characteristics and mental disorder: faris and Dunham revisited. Soc Sci Med.

[79] Van Os J (2004). Does the urban environment cause psychosis?. Br J Psychiatry.

[80] Dalack GW, Roose SP (1990). Perspectives on the relationship between cardiovascular disease and affective disorder. J Clin Psychiatry.

